# PombeX: Robust Cell Segmentation for Fission Yeast Transillumination Images

**DOI:** 10.1371/journal.pone.0081434

**Published:** 2013-12-06

**Authors:** Jyh-Ying Peng, Yen-Jen Chen, Marc D. Green, Sarah A. Sabatinos, Susan L. Forsburg, Chun-Nan Hsu

**Affiliations:** 1 Institute of Biomedical Informatics, National Yang-Ming University, Taipei, Taiwan, R.O.C.; 2 Department of Education and Research, Taipei City Hospital, Taipei, Taiwan, R.O.C.; 3 Center for Systems and Synthetic Biology, National Yang-Ming University, Taipei, Taiwan, R.O.C.; 4 Molecular and Computational Biology Program, University of Southern California, Los Angeles, United States of America; 5 Division of Biomedical Informatics, Department of Medicine, University of California, San Diego, La Jolla, United States of America; 6 Institute of Information Science, Academia Sinica, Taipei, Taiwan, R.O.C.; 7 Information Sciences Institute, University of Southern California, Marina del Rey, California, United States of America; Cancer Research UK London Research Institute, United Kingdom

## Abstract

*Schizosaccharomyces pombe* shares many genes and proteins with humans and is a good model for chromosome behavior and DNA dynamics, which can be analyzed by visualizing the behavior of fluorescently tagged proteins *in vivo*. Performing a genome-wide screen for changes in such proteins requires developing methods that automate analysis of a large amount of images, the first step of which requires robust segmentation of the cell. We developed a segmentation system, PombeX, that can segment cells from transmitted illumination images with focus gradient and varying contrast. Corrections for focus gradient are applied to the image to aid in accurate detection of cell membrane and cytoplasm pixels, which is used to generate initial contours for cells. Gradient vector flow snake evolution is used to obtain the final cell contours. Finally, a machine learning-based validation of cell contours removes most incorrect or spurious contours. Quantitative evaluations show overall good segmentation performance on a large set of images, regardless of differences in image quality, lighting condition, focus condition and phenotypic profile. Comparisons with recent related methods for yeast cells show that PombeX outperforms current methods, both in terms of segmentation accuracy and computational speed.

## Introduction

Fission yeast (*S. pombe*) and humans share many orthologous genes required for the DNA damage response and maintenance of an intact genome [1]. We derived a set of yeast mutants from a collection of approximately 4,000 mutants [2], each of which contains two fluorescently tagged marker proteins: RPA-CFP and Rad52-YFP, in an attempt to identify mutations that create genome instability and change the distribution of these proteins. For each genotype (gene deletion) we acquired 3 to 5 sets of microscopic images with three channels: a transmitted illumination channel, a CFP channel (RPA-CFP/ssb1-CFP/rad11-CFP) and a YFP channel (Rad52-YFP/rad22-YFP). Details of this screen and the results will be presented elsewhere.

The challenge now is to analyze more than 20,000 image files (2 markers×3 images×∼4,000 genes) to characterize representative phenotypes, and ultimately construct DNA repair pathways by correlating these quantitative phenotypes with known gene networks. The first task in the high content analysis of these images is to accurately segment cell boundaries, which may be obscured by inhomogeneous focus and contrast across a single image, due to imperfect alignment of substrate and the focal plane. Also images acquired in multiple sessions by different people may exhibit variant focus qualities, resulting in variable thickness and intensity of the cell membrane in different images, and even different intensity gradients from background to cell interior. In this work, we present a robust cell segmentation system, PombeX, for *S. pombe* cells in transillumination images.

## Methods

**Figure 1:**
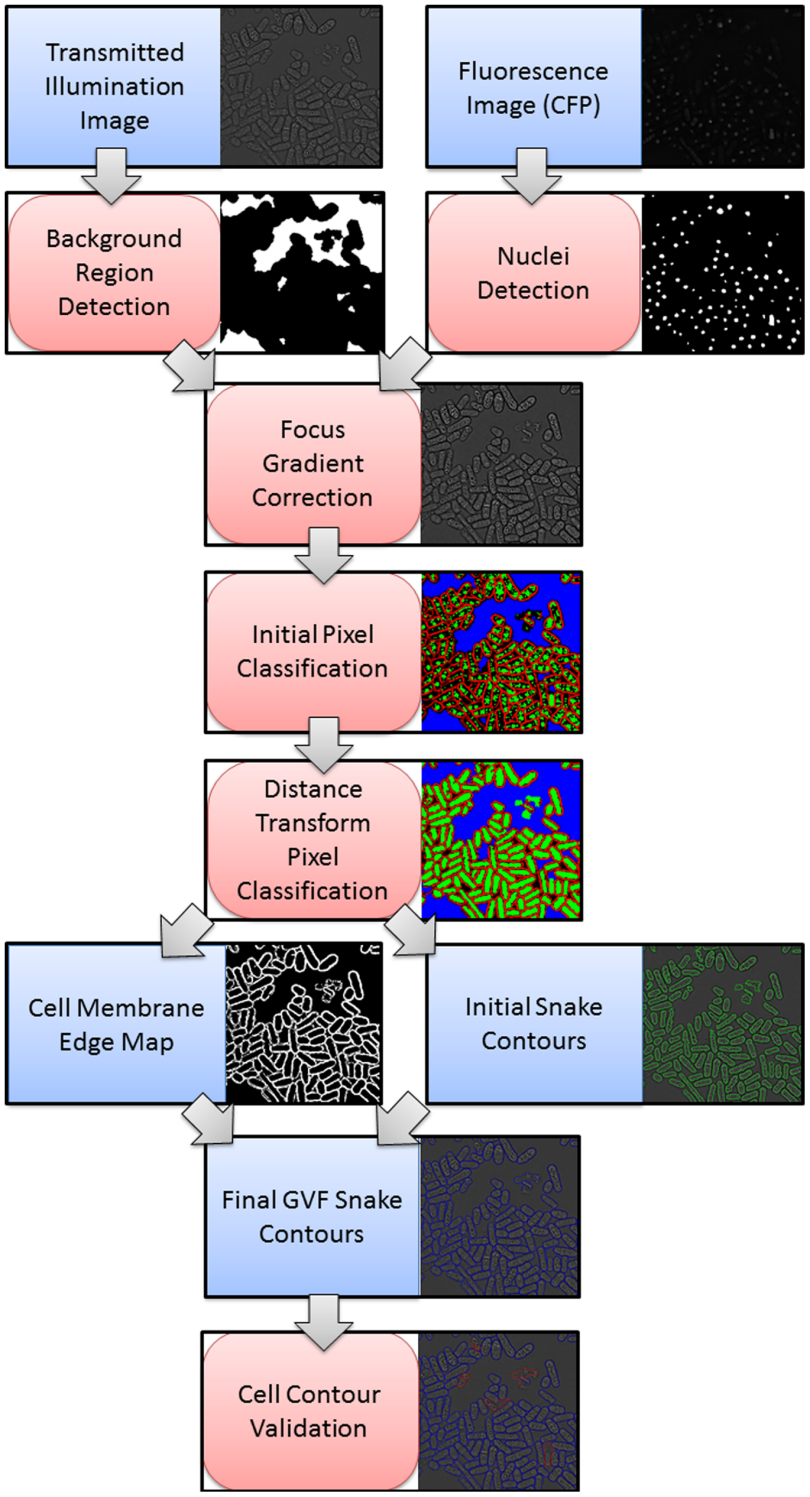
Overview of PombeX cell segmentation method.

Our pombe cell segmentation method consists of the following steps: First we identify cell nucleus and background regions, which are then used to adjust focus gradient differences and enhance contrast between cell interior, membrane and background in the transmitted light image. Next a distance transform-based pixel classification method identifies approximate cell interiors to form an initial cell contour, and an approximate cell membrane edge map is generated to be used in the gradient vector flow (GVF) snake model. After obtaining the final GVF snake contour, machine learning classifiers are trained and employed to validate the cell contours. Figure 1 presents an overview of our method, in the following we present details of each step.

### Nucleus and Background Region Detection

We first perform local shading correction of the transmitted illumination and fluorescent images by subtracting the original image by its average filtered image using a disk kernel of radius 40 pixels. Then we detect the background in transmitted illumination images by Otsu’s thresholding of its gradient image. The fluorescent signals are confined to the nucleus, so we further apply Otsu’s thresholding on the fluorescent images to obtain the final nucleus boundaries. From these we can then calculate the average intensity of nucleus regions *μ_N_* and background regions *μ_B_* in the transmitted illumination image, which are then used to correct for the relative focus gradient across different images. If no nuclear fluorescent images are available, we set *μ_N_* = *μ_B_* and skip the focus correction step.

Note that not all cells exhibit fluorescent signals in the nucleus, hence the intensity characteristics of the trans-illumination images are still needed to infer the position of non-fluorescent cells. Our system is able to correctly detect and segment most cells without requiring all cells to exhibit fluorescence.

### Focus Gradient Correction

**Figure 2:**
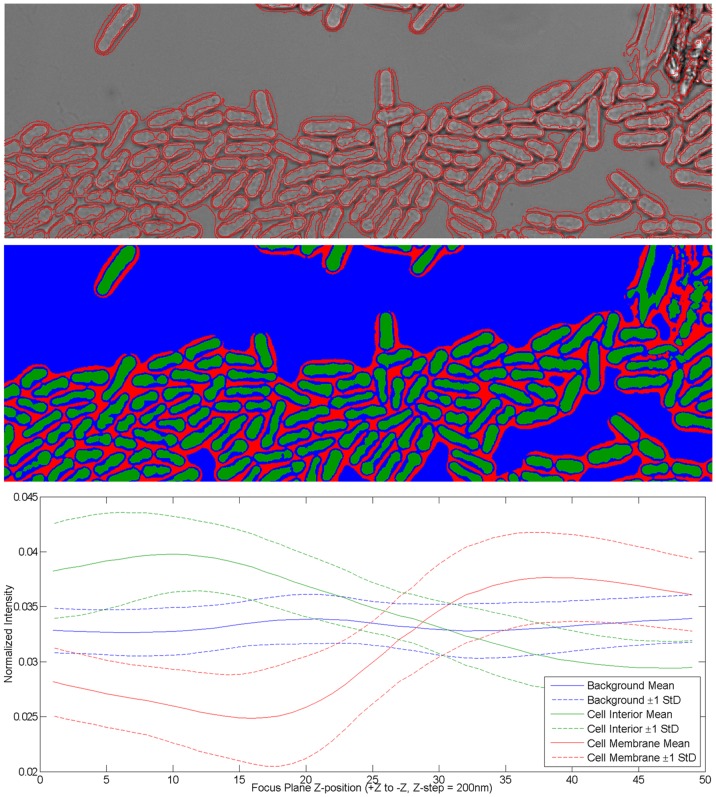
Analysis of pixel intensity variation of the same specimen imaged at different z-focus position. Top plot: original image at one z-position with red contours indicating k-means cluster boundaries. Middle plot: the same image with color-coded cluster membership for pixels. Bottom plot: Mean intensity of each pixel cluster through z-position.

To derive our focus gradient correction procedure, we imaged pombe cells using changing focus positions, and investigated the intensity changes when we vary the focus position from above the coverslip and downward towards the specimen, going past optimal focus. We performed *k*-means clustering (*K* = 3) of image pixels using their intensity values at different focus positions as features. The top and middle plots in Figure 2 show that pixels are automatically clustered into cell interior, cell membrane and background regions. These three regions undergo different intensity changes through varying z-position (Figure 2, bottom plot). If we only look at the middle range (z-step 16–34) where the defocusing is not too severe, there seems to be a symmetry between the mean intensity of cell interior and cell membrane about mean background intensity. Thus we formulate a model where the average background intensity stays constant at *μ_B_*, and the intensities of both the cell membrane and interior satisfies the relation
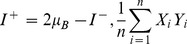
(1)where *I* ^+^ is the pixel intensity when positively defocused, and *I* ^−^ is the intensity when negative defocused.

Thus to perform focus gradient correction, for each image we first calculate the average intensity of nucleus regions *μ_N_* (representing cell interior intensity) and background regions *μ_B_* in the transmitted illumination image, and then linearly adjust the intensity in the whole image so that *μ_N_*–*μ_B_* matches a predefined value (for the entire dataset), complementing the image if necessary. This procedure also enhances the overall contrast of the image. After this correction, most images will have cell interior regions consistently brighter than the background, and the cell membrane consistently darker. This facilitates the subsequent classification of pixels into cell interior, cell membrane and background, which serves as a basis in determining initial snake contours for each cell, and the gradient vector flow (GVF) edge map.

Some of the transmitted illumination images exhibit a focus gradient caused by the specimen plane not entirely in parallel to the focal plane, resulting in the intensity characteristics of cell interior and membrane changing across a single image. Consequently, the global focus correction presented in the last paragraph only works for part of the image. To analyze these images, we assume that the specimen plane is flat, and use a bilinear model to approximate the distance to the focal plane at each pixel position,
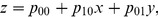
(2)where *z* can be positive or negative depending on whether the specimen point is above or below the focal plane, and *x*, *y* is the specimen point position in pixels. To approximate *z* values, we make a second assumption that the intensity difference of nucleus to background is linearly related to *z*, which is justified when the defocusing is not too severe as shown in Figure 2.

Our adaptive focus gradient correction algorithm can be stated as follows:

1. If the image has focus gradient, then

- Detect the location *x*, *y* of all nuclei and their intensity difference to background *z*.
- Fit a bilinear model (eq. 2) to *x*, *y*, *z*.
- For every pixel where *z* < 0, complement the pixel intensity *i* with respect to the average background intensity *μ_B_* by *j*  =  2*μ_B_*–*i*.

2. Globally and linearly adjust the intensity of the whole image so that the difference between average nucleus intensity (of the corrected image) and background intensity equal a predefined value.

**Figure 3:**
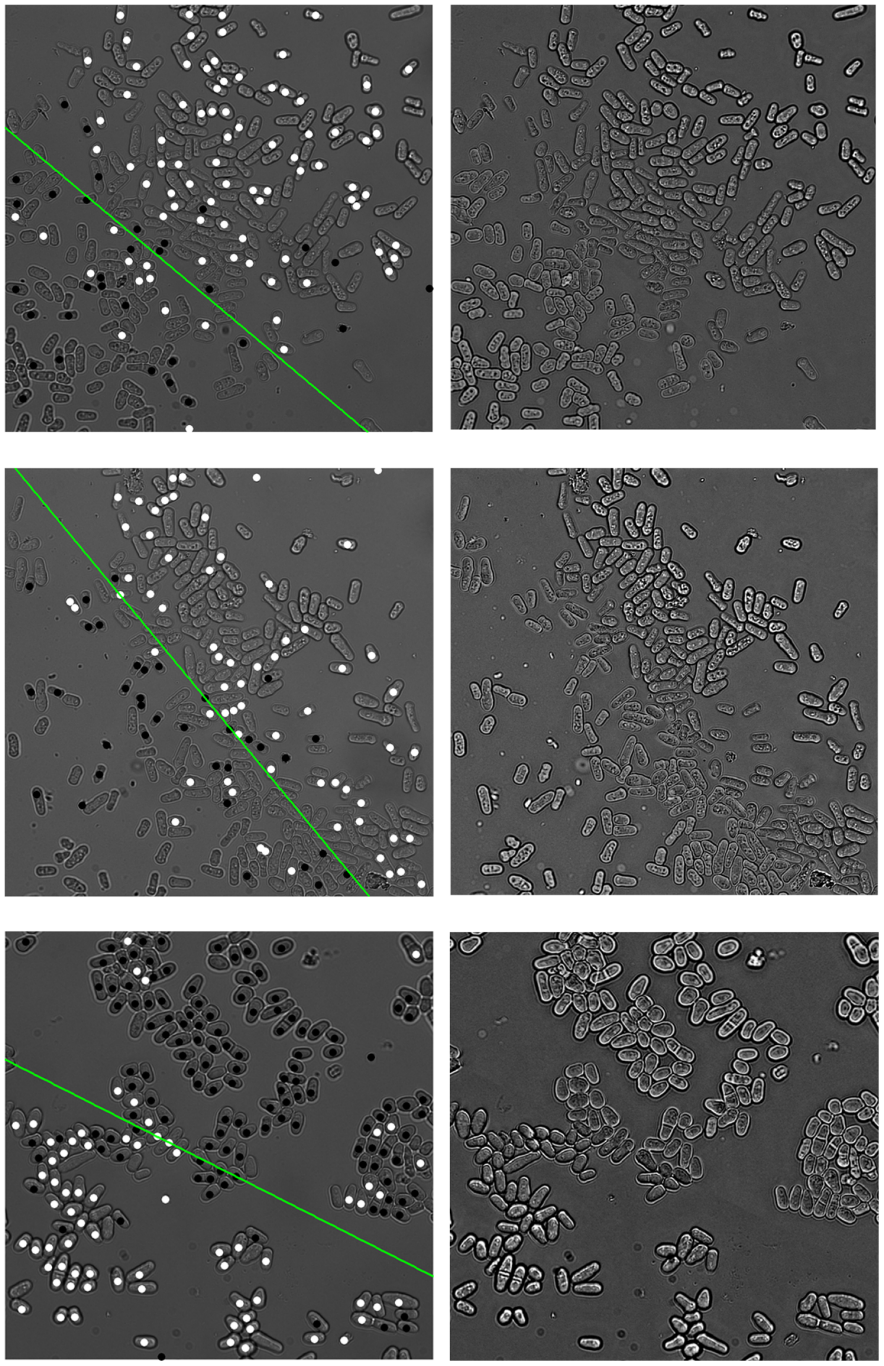
Examples of images before (left) and after (right) adaptive focus gradient correction and contrast enhancement. In the left panel the original images are overlaid with nucleus centroids (black and white spots) and the estimated *z* = 0 line (green line, where specimen plane intersects focal plane). For the nucleus centroids, black means its average intensity is lower than background, and white means it is brighter than background.

Figure 3 shows how our method is applied and examples of corrected images. The original image contains a focus gradient effect where the average intensity of cell interior and membrane varies across the image and is higher than background intensity on one side of the image and lower on the other. After adaptive focus gradient correction, we can see that all image regions with sufficient contrast now consistently have the cell interior brighter than the background, and cell membrane darker, although the final position of detected cell membrane would vary somewhat. Since the ultimate goal is to detect fluorescence belonging to a single cell, it is tolerable to allow small fluctuations in detected membrane positions.

### Cell Segmentation

We used active contour models [3] to obtain cell boundary contours. We first use adaptive thresholds [4] to classify pixels into four distinct sets: background, cell interior, cell membrane and ambiguous. Cell membrane pixels have intensity that are below an adaptive threshold calculated from all pixels darker than the background, whereas cell interior pixels have intensity greater than the average intensity of the nucleus region. Morphological close-opening and removable of isolated pixels were performed to smooth each region, then the remaining unclassified pixels *X* (ambiguous) were classified by a distance transform-based procedure (similar to watershed) that extends the background and cell interior regions to neighboring ambiguous regions:

1. Calculate distance transforms *D_b_* of background set *B*, *D_c_* of cell interior set *C*, and *D_m_* of cell membrane set *M*.

2.
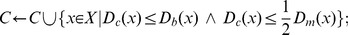


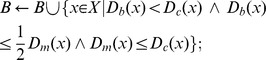
 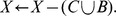


3. Repeat Steps 1 and 2 until *X* is unchanged.

**Figure 4:**
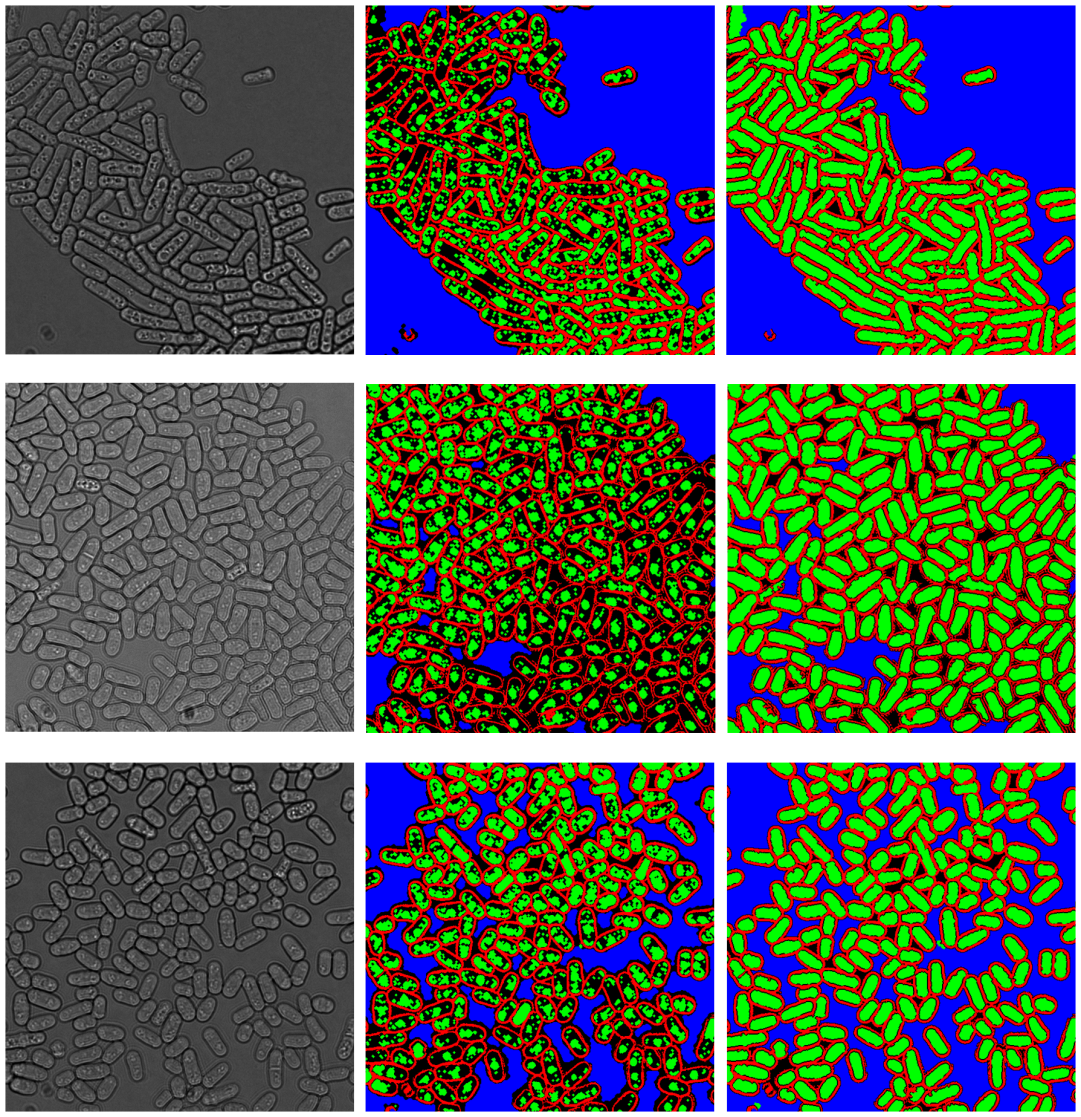
Example results of distance transform-based procedure. Original images (left), pixel classification before (middle) and after (right) distance transform-based procedure. Blue pixels indicate background, red is cell membrane and green is cell interior region.

Figure 4 shows some example results of this procedure. The initial snake contours are obtained from individual connected cell interior regions, while the cell membrane edge map is obtained from pixels classified as cell membrane.

Next we use the following snake model with energy functional *E_snake_* for contour **x**(*s*) = [*x*(*s*), *y*(*s*)], *s* ∈ [0,1]:
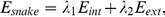
(3)where *λ*_1_, *λ*_2_ controls relative weighting between internal and external energies. The conventional internal energy of the snake contour is
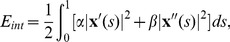
(4)where the weighting parameters *α* and *β* control the snake’s tension and rigidity, respectively. For the external image energy *E_ext_*, we used the gradient vector flow (GVF) snake [5]. Given an edge map *f*(*x*, *y*) derived from the image *I*(*x*, *y*), the GVF field **v**(*x*, *y*) = [*u*(*x, y*), *v*(*x*, *y*)] minimizes the energy functional
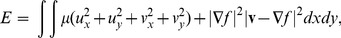
(5)where **v** denotes –∇*E_ext_*, and *f* is the cell membrane edge map obtained previously. The parameter values used are *λ*_1_ = 1, *λ*_2_ = 1, *α* = 0.03, *β* = 0.2 and *μ* = 0.8. The computation of the gradient vector flow (GVF) field from the edge map, and the snake deformation iterations is as described in [5].

### Cell Contour Validation

To further increase the robustness and efficiency of the system, we also employ machine learning classifiers to automatically validate the final snake contours. Specifically, we aim to detect errors such as merging of two or more cells, partial segmentation, and false detection of background or artifacts. Manually labeled training samples are generated for each type of error, and we used support vector machines with radial basis function to learn and classify partial segmentation and merging errors. False detection of background and artifact are relatively rare and well distinguished from normal cells, thus a simple binary classification tree is trained and used for this error type. Cells intersected by the image boundary share the same characteristics as a partially segmented cell, and are treated equivalently by our classifier.

## Results

**Figure 5:**
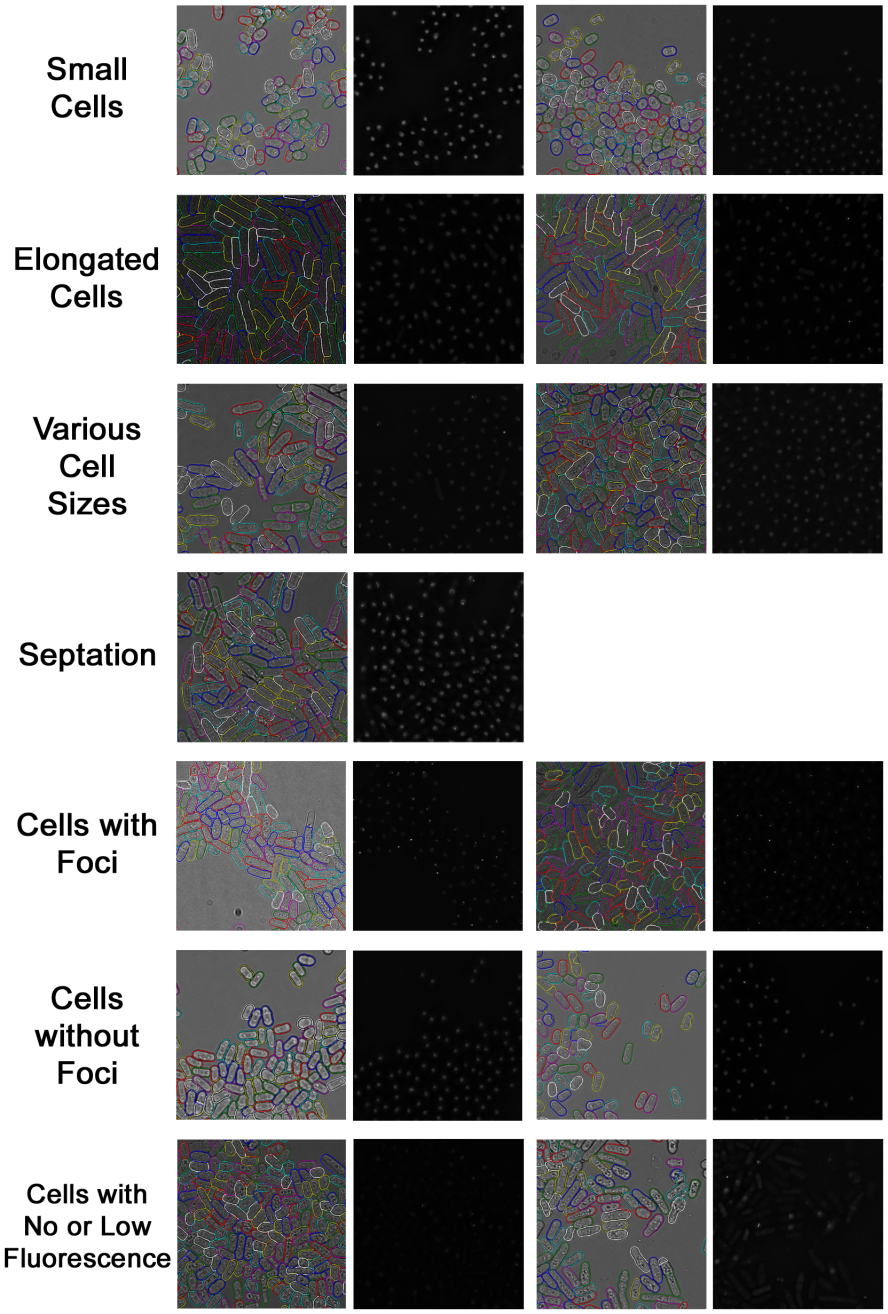
Representative segmentation results on various pombe mutants. For each image pair, trans-illumination images with randomly colored cell contours are shown on the left, and the corresponding RPA-CFP images on the right.

We applied PombeX to multi-channel images of *S. pombe*. The whole data set contains about 4,000 mutant genotypes, each with at least three sets of transmitted illumination (bright field), Rad52-YFP and RPA-CFP images. Our system is able to correctly segment a majority of cells in almost all images of sufficient quality. The performance is consistent over a wide variety of focal distance, field brightness, relative contrast and phenotypic characteristics. Moreover, some images contain dead cells with autofluorescence or no fluorescence, exhibited as enlarged nuclei or no nuclei, but our system is still able to correctly segment the transmitted illumination image given only a subset of nuclei positions. Figure 5 shows PombeX segmentation of a variety of phenotypes, including small and elongated cell shapes, cells in septation, and cells with various fluorescent signal profiles.

**Figure 6:**
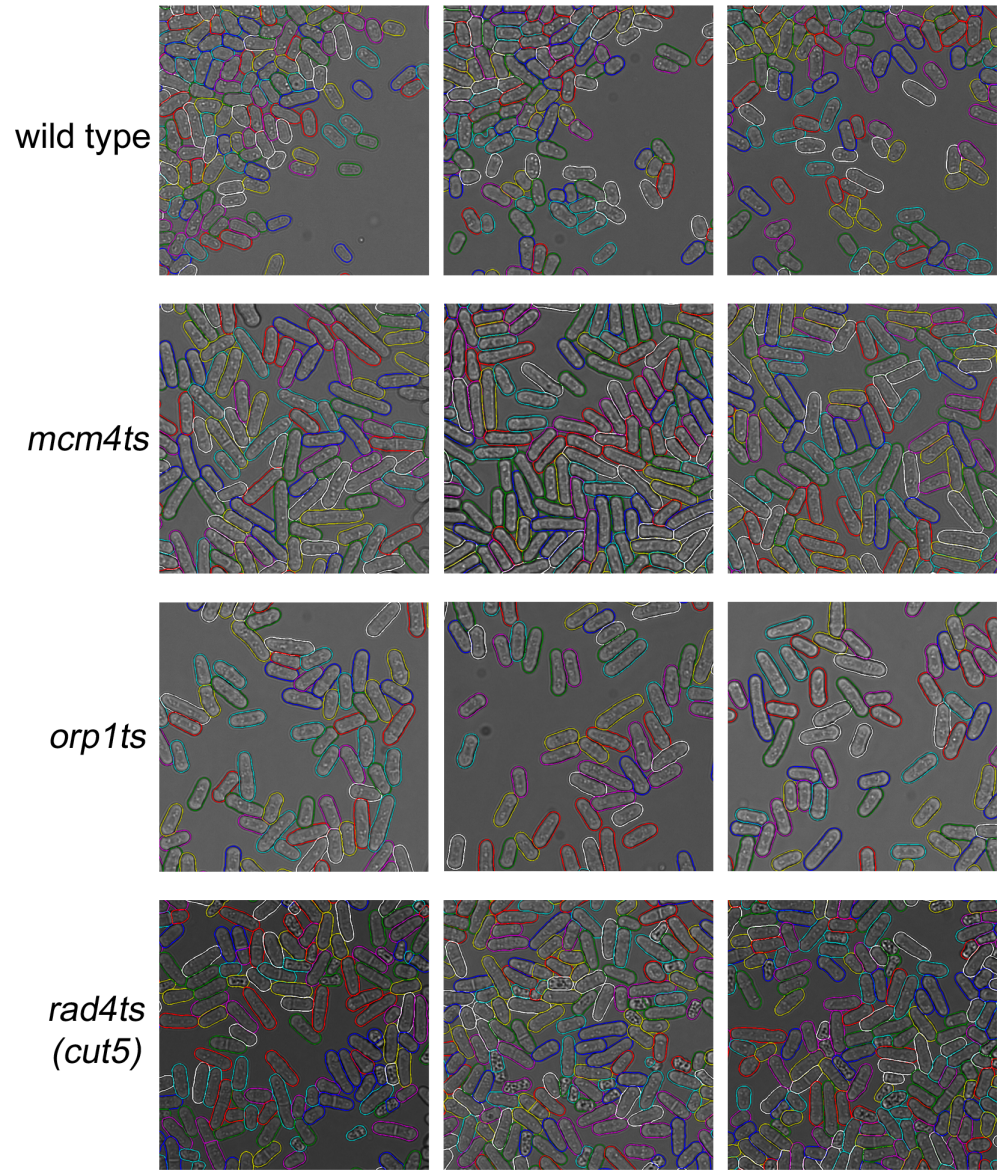
Segmentation results on well-known phenotypes. Cell contours are colored randomly.

To further validate our method, we used heat treatment to induce phenotypic changes in mutant cells, and test the performance of PombeX on these classic phenotypes. In Figure 6, asynchronous wild type cells were taken from a late-exponential culture before treatment, causing a slightly smaller distribution of asynchronous wild type cell lengths. *mcm4ts* cells are an example of a classical cell division cycle (cdc) phenotype, which elongate during temperature treatment (*cdc21-M68* allele [6]). In contrast, *orp1ts* cells (*orp1-4* allele, the human ORC1 homologue [7]) do not replicate their DNA at 36°C, causing an accumulation of different cell sizes. The *rad4ts* (*cut5*) cells fail at both DNA replication and in the DNA damage checkpoint (*rad4-116* allele [8]). As a result they form small cells with a septum slicing through DNA in the centre of the cells, causing a cell untimely torn (cut) morphology. From the results we can see that PombeX can produce good segmentations for all of these phenotypes.

Finally, we performed quantitative evaluation and comparison with two recent automated segmentation algorithms for yeast cells, CellStat [9] and CellSerpent [10], using a set of hand produced gold standard segmentations of pombe cells, representing different image acquisition conditions and quality. CellStat first finds candidate cell centers using a variation of the circular Hough transform, and then cell contours surrounding the candidate cell centers are extracted by directional derivatives and curve matching. CellSerpent used nonlinear degenerate elliptic smoothing to remove small features that are not cell membranes, generating an edge penalization image. Starting from local maxima in the edge penalization image, active contour models are used to find the final cell contours.

**Figure 7:**
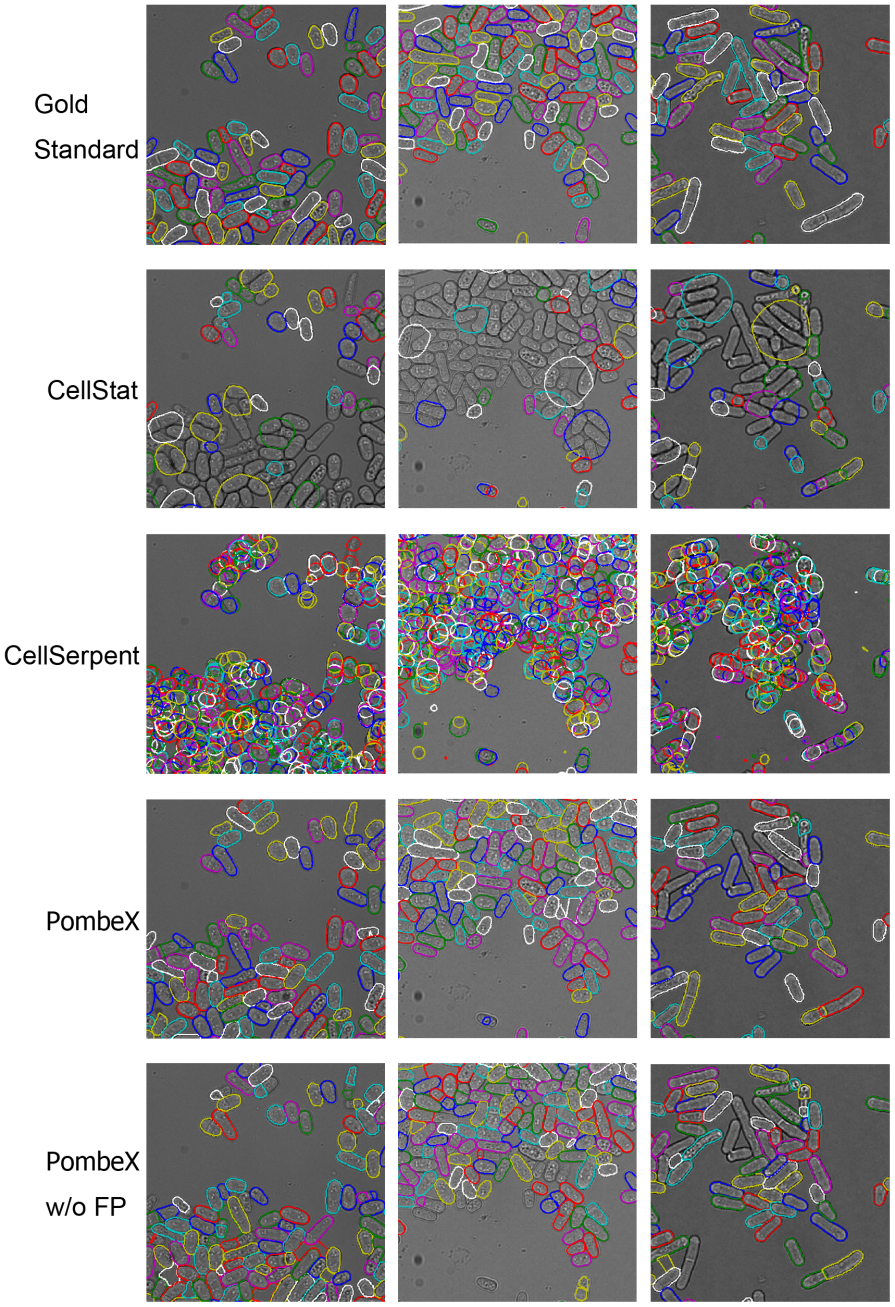
Segmentation method comparison. Contours of different cells are colored randomly.

We evaluated the percentage of cells detected, the segmentation accuracy of the final snake contours, and the automatic error classifier. The whole set of 64 gold standard images contain a total of 16,170 pombe cells, averaging about 253 cells per image. The quantitative performances of CellStat, CellSerpent and PombeX are shown in Table 1. Figure 7 shows representative results from all methods. We tuned the parameters of CellStat and CellSerpent to optimize their performance on our data set. The parameter settings for CellStat are: Sigma Mult. Factor: 3 (default 7.5), Max Eccentricity: 15 (default 1.5), Max Area: 15000 (default 1500), and Max Radius to Centre: 100 (default 25). The parameter settings for CellSerpent are: Inflating force *β* = 2.24 (default *β* = 2). Since CellStat and CellSerpent did not use fluorescent signals, we also tested a version of our method that do not make use of the fluorescent channel images (listed as PombeX w/o FP in Table 1).

**Table 1:** Quantitative performance results of yeast segmentation algorithms.

Method	%Cells	%Cells	# of Contours	PPV^3^	CPU
	Detected^1^	Segmented^2^	Generated		time^4^
CellStat [9]	73.0%	10.2%	9164	18.8%	2382.5
CellSerpent [10]	99.8%	32.0%	104122	11.3%	1887.7
PombeX	97.0%	86.4%	18125	86.9%	135.7
PombeX w/o FP	94.5%	70.8%	19594	71.1%	125.0

^1^ Relative to all cells.

% pixel mismatch compared to gold standard cell contours, relative to all cells.^2^ Defined as the percentage of cell contours with less than 10

^3^ Relative to all contours.

CPU@3.40 GHz, 4 GB RAM).^4^ Average CPU time per image, measured using a regular desktop PC (Windows 7, Matlab R2011a, Intel(R) Core(TM) i7-2600

Our automatic cell contour validation system has an overall classification accuracy of 86.4%, with sensitivity 85.7% and specificity 85.4%. After erroneous cell contours are removed by our cell contour validation classifier, the remaining cell contours contain 97.8% true positives (precision, *i.e.* % relative to all detected cell contours). This shows that the final cell contours generated by our method after automatic validation are very reliable. For large scale high-throughput applications with huge amounts of data, in order to minimize the need for human intervention, the high reliability and robustness achieved by our system is valuable.

CellStat and CellSerpent were primarily designed for bright field images of *Saccharomyces cerevisiae* cells, which are round cells with good border contrast. This may in part account for their poor performance for correctly segmenting elongated pombe cells, since the final generated contours are biased toward circular shapes. CellSerpent was designed for images of crowded round budding yeast cells, and the elongated fission yeast cells in our images may be over-segmented into multiple round cells. On the other hand, CellStat assumes that each cell has a major part of its border touching the background, so cell misses and incorrect merging occurs for clustered or elongated cells.

## Discussion

There are some recent works on cell image segmentation [11]–[13], which applied active contour models such as parametric snakes or level sets. Most of these methods are designed for phase-contrast images with higher contrast between cell membrane and background, and are designed with objective functions that depend only on relative brightness. Suzuki *et al.* [14] segmented and quantitatively characterized budding and fission yeast cells, but their images are either stained with cell wall fluorescence (which is trivial to segment) or phase contrast images. They did not address the more complex problem of detecting and segmenting cells from ordinary bright field images. Tscherepanow *et al.* [15] analyzed images very similar to the ones presented here (bright field images of Sf9 cells), but one of the main drawbacks of their method is its inability to deal with images with different focus properties, which are predominant in our dataset, and for which we have developed both global and locally adaptive methods of focus gradient correction.

The adaptive focus gradient correction uses a bilinear model to estimate the intersection between the specimen plane and the objective focal plane, but this model may be too simplistic resulting in many cells not being focus corrected. It may be possible to improve this by explicitly using margin maximization methods, such as support vector machines, to find the exact boundaries between positively and negatively defocused regions. The adaptive focus correction algorithm is designed so that even when only a part of the image is complemented, *C* ^0^ continuity of the pixel intensities is still maintained because the complement is taken with the background intensity as center. But sometimes cells cut by the *z* = 0 boundary would still be severely distorted. This may require warping the boundary so that it only crosses the background pixels, though this solution is only feasible for images with cells not too densely packed.

The proposed segmentation system, PombeX, can be applied to a variety of experimental questions. For our screen, the next steps will be to characterize the fluorescent signals both within the nucleus and cytoplasm, as well as cell morphology. This will require identification and quantification of individual puncta at normal fluorescent intensity. Fluorescence that enters the cytoplasm can be used to automatically identify dead or dying cells. Additional information that may be culled from these data will include cell morphology to identify cell cycle stage. Finally, changes in cell dimension including average length or length × width dimensions can be quantified.

In conclusion we have developed a robust cell segmentation system for *S. pombe* cells that uses nucleus protein fluorescence to correct varying focus and contrast in the transmitted illumination image, combined with active contour segmentation and robust automatic contour validation. This system can be applied to similar bright-field microscopy images with or without corresponding fluorescence signal within the cell nucleus or cytoplasm, and can in principle be extended to deal with multiple cell types and image modalities.
